# Complete Genome Sequence of Staphylococcus haemolyticus Type Strain SGAir0252

**DOI:** 10.1128/genomeA.00229-18

**Published:** 2018-05-10

**Authors:** Balakrishnan N. V. Premkrishnan, Ana Carolina M. Junqueira, Akira Uchida, Rikky W. Purbojati, James N. I. Houghton, Caroline Chénard, Anthony Wong, Sandra Kolundžija, Megan E. Clare, Kavita K. Kushwaha, Deepa Panicker, Alexander Putra, Nicolas E. Gaultier, Cassie E. Heinle, Vineeth Kodengil Vettath, Daniela I. Drautz-Moses, Stephan C. Schuster

**Affiliations:** a Singapore Centre for Environmental Life Sciences Engineering, Nanyang Technological University, Singapore; b Asian School of the Environment, Nanyang Technological University, Singapore

## Abstract

Staphylococcus haemolyticus is a coagulase-negative staphylococcal species that is part of the skin microbiome and an opportunistic human pathogen. The strain SGAir0252 was isolated from tropical air samples collected in Singapore, and its complete genome comprises one chromosome of 2.63 Mb and one plasmid of 41.6 kb.

## GENOME ANNOUNCEMENT

The Staphylococcus genus is associated with several bacterial infections in humans and other animals and is described as part of the human skin microbiome (1). Staphylococcus haemolyticus is included in the coagulase-negative staphylococci (CoNS) group and is one of the most important human-associated pathogens (2) belonging to the Staphylococcus genus. S. haemolyticus has been implicated in native valve endocarditis, septicemia, peritonitis, and urinary tract infections, and is occasionally associated with wound, bone, and joint infections (3).

Air samples were collected in Singapore using an Andersen single-stage impactor (SKC BioStage). This air sampler collects bioaerosol particles at a flow rate of 28.3 liter/min. Viable air particles were collected on Trypticase soy agar (TSA; Becton, Dickinson) media plates placed below the sampler and incubated overnight at 30°C. Further isolation was conducted in Luria-Bertani (LB) broth incubated at room temperature overnight. DNA was extracted using a Wizard Genomic DNA purification kit (Promega), following the manufacturer’s protocol. A whole-genome shotgun (WGS) library was prepared with the SMRTbell template prep kit 1.0 (Pacific Biosciences), using a 15-kb cutoff. Single-molecule real-time (SMRT) sequencing was performed on a PacBio RS II (Pacific Biosciences) sequencer. Additional WGS libraries were prepared with a TruSeq Nano DNA library preparation kit and sequenced on an Illumina MiSeq instrument to generate 300-bp paired-end reads. The total number of long reads obtained with the PacBio RSII platform was 23,264 (71.33-fold coverage), whereas the Illumina MiSeq platform provided 653,569 (121.27-fold coverage) short reads.

*De novo* genome assembly of long reads was performed using the Hierarchical Genome Assembly Process version 3 (HGAP3) implemented in the SMRT analysis algorithm version 2.3.0 (4). Different stages of the process involved preassembly, *de novo* assembly with Celera Assembler, and polishing with Quiver (4). Illumina reads were used to improve the genome contiguity, with the help of the error correction software Pilon version 1.16 (5). The complete circular genome contained two contigs, consisting of one chromosome of 2,632,932 bp (71.3-fold coverage) and the plasmid pSGAir0252 of 41,662 bp (18.63-fold coverage). The overall G+C content was 32.78%.

The Phylum-specific Automated Phylogenomic Inference Pipeline (Phyla_AMPHORA) (6) predicted 100% identity at the species taxonomic level. Further taxonomic assignment was investigated with the average nucleotide identity (ANI) method, using the Microbial Species Identifier (MiSI) (7), which showed 99.56% identity with the available reference genome sequence of Staphylococcus haemolyticus JCSC1435 (NCBI reference sequence number NC_007168).

Multilevel genome annotation was conducted by the NCBI Prokaryotic Genome Annotation Pipeline (PGAP) (8). The complete genome consists of 2,528 genes, of which 2,445 were protein-coding genes, 19 rRNA subunits (7 genes for 5S, 6 for 16S, and 6 for 23S), 60 tRNAs, and 4 noncoding RNAs. A total of 108 pseudogenes were predicted. Functional annotation using the Rapid Annotations using Subsystems Technology (RAST) server (9–11) revealed that 75 of the genes were associated with virulence, disease, and defense, 140 genes with RNA metabolism, 196 genes with carbohydrate metabolism, and 259 genes with metabolism of amino acids and derivatives.

### Accession number(s).

The complete genome sequence of Staphylococcus haemolyticus strain SGAir0252, including the plasmid pSGAir0252, is available in GenBank under the accession numbers CP025031 (chromosome) and CP025033 (pSGAir0252).
