# Correction for Premkrishnan et al., “Complete Genome Sequence of Staphylococcus haemolyticus Strain SGAir0252”

**DOI:** 10.1128/MRA.00736-19

**Published:** 2019-08-01

**Authors:** Balakrishnan N. V. Premkrishnan, Ana Carolina M. Junqueira, Akira Uchida, Rikky W. Purbojati, James N. I. Houghton, Caroline Chénard, Anthony Wong, Sandra Kolundžija, Megan E. Clare, Kavita K. Kushwaha, Deepa Panicker, Alexander Putra, Nicolas E. Gaultier, Cassie E. Heinle, Vineeth Kodengil Vettath, Daniela I. Drautz-Moses, Stephan C. Schuster

**Affiliations:** a Singapore Centre for Environmental Life Sciences Engineering, Nanyang Technological University, Singapore; b Asian School of the Environment, Nanyang Technological University, Singapore

## AUTHOR CORRECTION

Volume 6, no. 19, e00229-18, 2018, https://doi.org/10.1128/genomeA.00229-18. Page 1: The title should appear as given in this correction.

